# Three synchronous lesions with different historical types diagnosed by endoscopic submucosal dissection in one patient

**DOI:** 10.1055/a-2161-3653

**Published:** 2023-09-21

**Authors:** Xue Chen, Benyan Zhang, Qi Sun, Xi Chen

**Affiliations:** 1 Department of Gastroenterology, Shanghai Jiao Tong University Medical School Affiliated Ruijin Hospital, Shanghai, China; 2 Department of Pathology, Shanghai Jiao Tong University Medical School Affiliated Ruijin Hospital, Shanghai, China

A 69-year-old man underwent gastroscopy owing to intermittent abdominal distension for over 4 months. The gastroscopy revealed two distinct lesions in the lower stomach body, which was highly atrophied (O-3) 1 , and the background mucosa was infected with *Helicobacter pylori.* Lesion 1, labeled as 0-Is + IIa 2 , measured 40 × 20 mm and had a nodular mixed-type appearance on the posterior wall of the stomach body ( Fig. 1 a, b ). Lesion 2, labeled as 0-IIb, measured 15 × 10 mm and was adjacent to lesion 1 on the oral side ( Fig. 2 a ). Biopsy pathology of both lesions showed atypical cells.

**Fig. 1:**
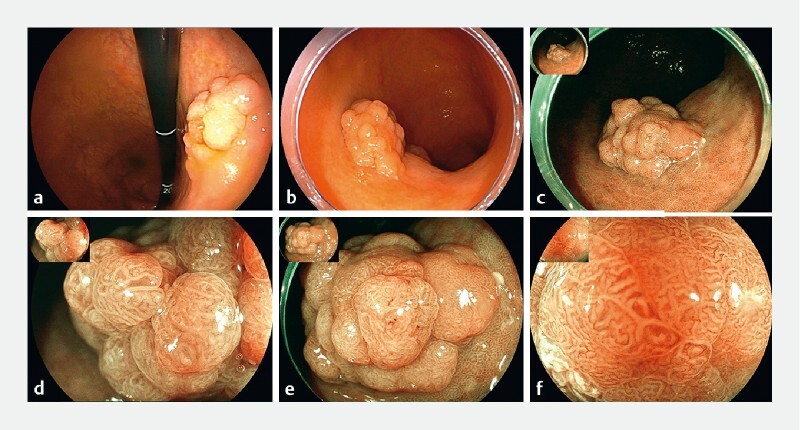
Features of lesion 1 under white light endoscopy and magnifying endoscopy with blue-laser imaging (ME-BLI).

**Fig. 2:**
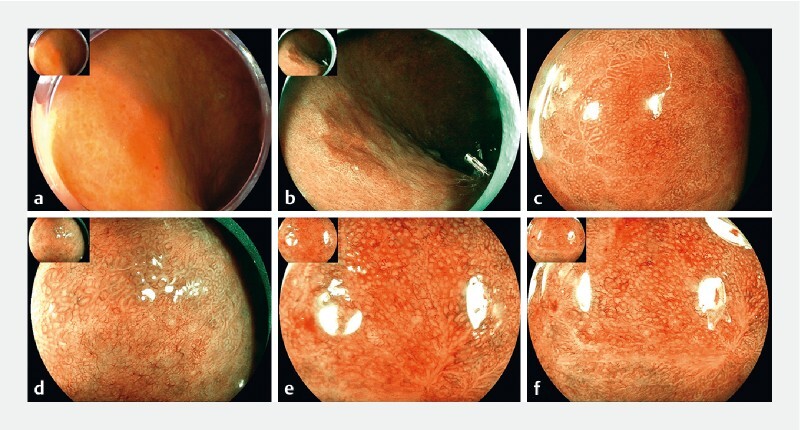
Features of lesion 2 under white light endoscopy and ME-BLI.

Further investigation using magnifying endoscopy with blue-laser imaging (ME-BLI) revealed that lesion 1 had a distinct boundary and mimicked a colonic laterally spreading tumor with a villous surface pattern ( Fig. 1 d, e ). ME-BLI also revealed that the area of the lesion presenting noticeable redness had an intensive and irregular vascular pattern ( Fig. 1 f ). Lesion 2 also had a distinct boundary and presented a brownish area. ME-BLI further revealed an irregular vascular pattern and white globe appearance ( Fig. 2 d, e ). Both lesions were removed completely by endoscopic submucosal dissection (ESD). The histological diagnosis was intestinal adenoma with partial high-grade intraepithelial neoplasia for lesion 1 and crawling-type adenocarcinoma 3 (tub2) for lesion 2 ( Fig. 3, Fig. 4 b, c ).

**Fig. 3:**
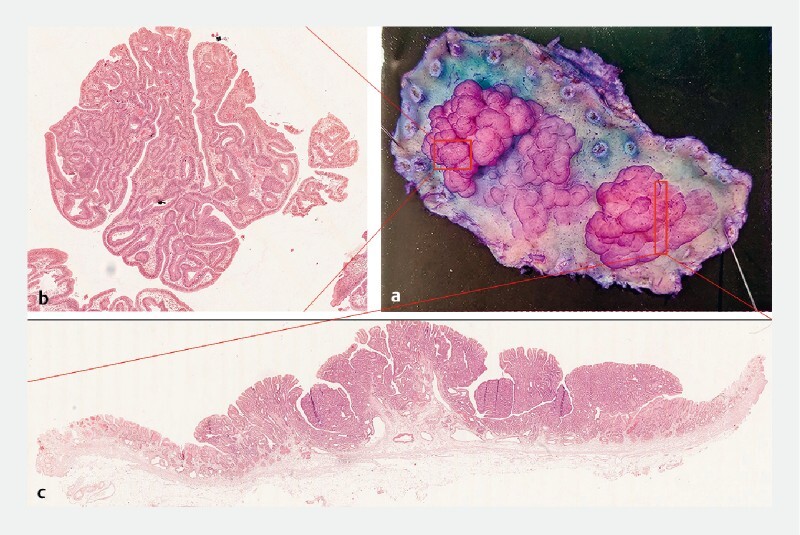
Postoperative specimen and hematoxylin and eosin (H&E) stain of lesion 1. **a** Endoscopic submucosal dissection specimen. **b** H&E stain of the red area. **c** H&E stain of the anal side.

**Fig. 4:**
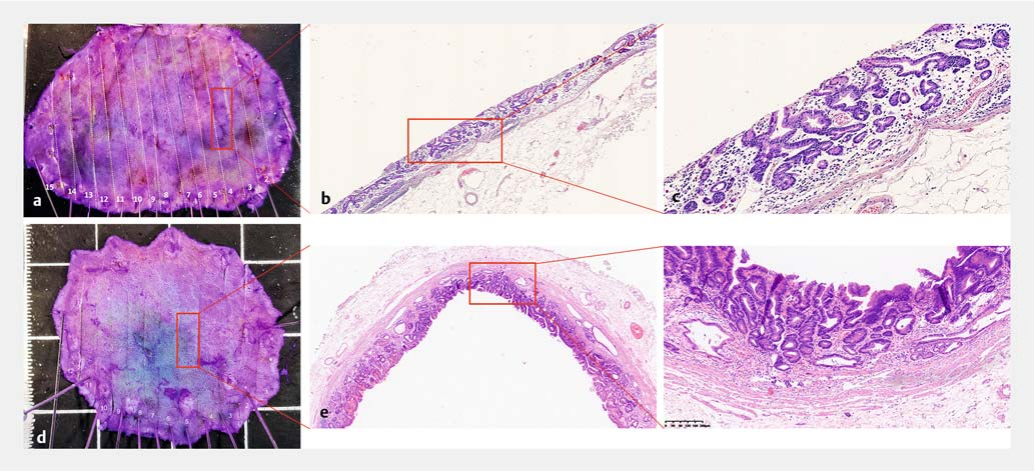
Postoperative specimen and H&E stain of lesions 2 and 3.

The patient underwent a follow-up gastroscopy after 10 months, which revealed a 15 × 10-mm 0-IIc lesion ( Fig. 5 ) with a clear boundary in the gastric antrum. Lesion 3 showed light redness, and further ME-BLI revealed increased density of the glandular ducts with an irregular surface and vascular pattern ( Fig. 5 d, e ). It was also removed by ESD and the final diagnosis was well-differentiated tubular adenocarcinoma (tub1) ( Fig. 4 e, f ).

**Fig. 5:**
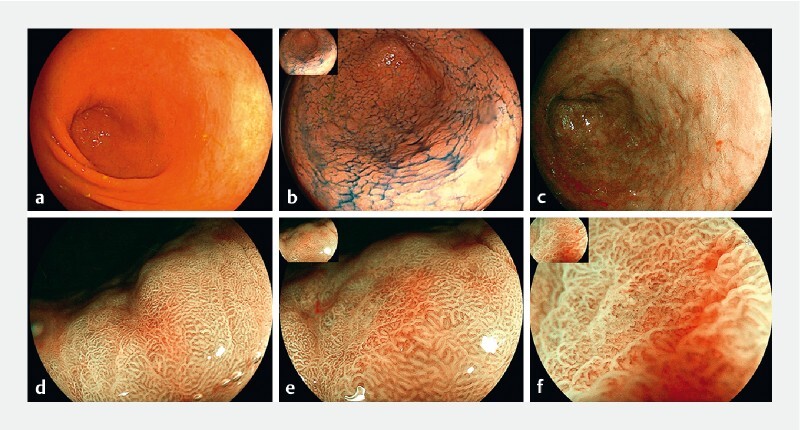
Features of lesion 3 under white light endoscopy and ME-BLI.

This case highlights the detection of three synchronous gastric lesions with different pathologic types ( Video 1 ). Each one had a different macroscopical appearance.

**Video 1** Three synchronous lesions with different historical types diagnosed by endoscopic submucosal dissection in one patient.


Endoscopy_UCTN_Code_CCL_1AB_2AD_3AB

## Notes

Endoscopy E-Videos

https://eref.thieme.de/e-videos
*E-Videos* is an open access online section of the journal *Endoscopy* , reporting on interesting cases and new techniques in gastroenterological endoscopy. All papers include a high-quality video and are published with a Creative Commons CC-BY license. Endoscopy E-Videos qualify for HINARI discounts and waivers and eligibility is automatically checked during the submission process. We grant 100% waivers to articles whose corresponding authors are based in Group A countries and 50% waivers to those who are based in Group B countries as classified by Research4Life (see: https://www.research4life.org/access/eligibility/ ). This section has its own submission website at https://mc.manuscriptcentral.com/e-videos
